# Observations on the Treatment of Hooping-Cough, and on the Use of Sulphate of Quinine in That Disease

**Published:** 1829-07

**Authors:** 


### HOOPING-COUGH.

*Observations on the Treatment of Hooping-Cough, and on the Use of Sulphate of Quinine in that Disease.* By a SURGEON.

In the beginning of 1828, on a homeward voyage from India, the hooping-cough prevailed in the ship of which I was surgeon. The number of children on board was seventeen, all of whom, with the exception of a girl who had had it before, were affected. The greatest age was eight years, and the youngest (if I mistake not) thirteen months. The contagion was communicated by a girl of four years, who was labouring under its influence at the period of our embarkation, (Jan. 6.) The disease was severest with the younger, while the older suffered comparatively little. There was nothing new, as far as I observed, in the appearances which it exhibited; but, as I employed a remedy from which I think very considerable benefit was derived, and which I am not aware has hitherto been much, if at all, in practice, the result may not, perhaps, be unacceptable to the profession.

When unequivocal symptoms of the disease appeared, doses of ipecacuanha, according to the age of the patient, were given night and morning, so as to produce full vomiting. In the intervening time, a mixture of antimonial wine, laudanum, and sulphate of quinine, made into a draught with syrup and water, was given thrice a day, at intervals of five hours. The dose for a child of two years was three drops of the antimonial wine, one of laudanum, and half a grain of quinine. When the first, or contagious stage, was over, the quantity of the two former was diminished, while the latter was increased. Burgundy-pitch plasters were applied to the breast, and between the scapulæ. The bowels were kept moderately open by calomel and rhubarb; the diet was light and nutritive. This treatment was generally successful in about a month.

There was an interesting boy of three years who suffered extremely. The convulsive paroxysms were violent, and the quantity and tenacity of the mucus such as threatened suffocation. He was reduced to such a degree, that (to use his nurse's phrase) he was a mere "bag of bones:" yet, by a steady perseverance in the above treatment, his recovery, though late, was yet complete. Several expedients to divert his attention, by play, toys, &c., were of use as auxiliaries The quinine in the second stage was decidedly beneficial; and it is in this stage, where the disease is supposed to remain in the system merely from the power of habit, that the exhibit of tonics, and above all the quinine, is indicated.

I was induced to make trial of this medicine from the great approbation with which Dr. Cullen mentions the virtues of Peruvian bark in this disease. "I consider the use of this medicine," says he, "as the most certain means of curing the disease in its second stage; and, when there has been little fever present, and a sufficient quantity of the bark given, it has seldom failed of soon putting an end to the disease."* In the cases that came under my obser-

* Cullen's Works, by Thomson, vol. ii. p. 463.

vation, there was little or no fever; and I should think, from the small bulk and the soluble nature of the quinine, that a sufficient quantity can be given, without the inconveniences attending the exhibition of the bark.

I have said that the quinine, in the second stage, was decidedly beneficial: it certainly appeared to me so; yet, perhaps, I ought to qualify the expression. In estimating the effect produced on diseases by remedies, it is difficult to determine with precision the exact share which these have, apart from adventitious circumstances, in bringing about a favorable termination. In the present instance, the state of the atmosphere appeared to exercise considerable influence over the disease. During moist, hazy weather, the expectoration was more copious and viscid, and difficult of separation. When the air was hot and dry, it was scanty, the cough more distressing, and in one or two instances streaked with blood. Between the tropics, and during the prevalence of the trade-winds, when the weather was fine and clear, it was particularly mild. How much we are to attribute to the state of the atmosphere, I know not: one thing, however, will, I think, be granted, that the constant succession of climate that is experienced during an Indian voyage will rather have a salutary than an injurious effect upon the disease.

Should the use of the quinine in hooping-cough prove efficacious in the hands of other practitioners, I shall feel ~~happy~~. It deserves, at least, a fair trial; and it is exempt alike from danger and inconvenience.

